# A literature review of a meta-analysis of BRAF mutations in non-small cell lung cancer

**DOI:** 10.1097/MD.0000000000034654

**Published:** 2024-02-23

**Authors:** Clint Taonaishe Chimbangu, Li Xi, Zhou Ya, Zhao Jiayue, Meng Xiao, Wang Ying, Yu Xingxu, Xiaomei Liu

**Affiliations:** a Jinzhou Medical University, Liaoning, Jinzhou, China; b Department of Oncology, the First Affiliated Hospital of Jinzhou Medical University, Liaoning, Jinzhou, China.

**Keywords:** BRAF mutation, BRAFV600E mutation, females, lung cancer, nonsmokers, NSCLC

## Abstract

**Background:**

The research on the relationship between the Braf Proto-oncogene (BRAF) mutation and lung cancer has generated conflicting findings. Nevertheless, there is an argument suggesting that assessing the BRAF status could offer benefits in terms of managing and prognosing individuals with non-small cell lung cancer (NSCLC). To present a comprehensive overview of this subject, we undertook an up-to-date meta-analysis of pertinent publications.

**Methods:**

We conducted an extensive literature search utilizing Medical Subject Headings keywords, namely “BRAF”, “mutation”, “lung”, “tumor”, “NSCLC”, and “neoplasm”, across multiple databases, including PubMed, EMBASE, ISI Science Citation Index, and CNKI. For each study, we calculated and evaluated the odds ratio and confidence interval, focusing on the consistency of the eligible research.

**Results:**

The meta-analysis unveiled a noteworthy correlation between BRAF mutation and lung cancer. No significant evidence was found regarding the connection between smoking and staging among individuals with BRAF mutations. Furthermore, a substantial disparity in the rate of BRAF mutations was observed between males and females.

**Conclusion:**

Our meta-analysis revealed a significant correlation between BRAF mutations and NSCLC. Moreover, we observed a higher incidence of BRAF lung mutations in females compared to males. Additionally, the BRAFV600E mutation was found to be more prevalent among female patients and nonsmokers.

## 1. Introduction

Lung cancer, the leading cause of cancer-related deaths worldwide, predominantly presents as non-small cell lung cancer (NSCLC), which is associated with a poor prognosis and a low 5-year survival rate.^[1]^ Traditional therapeutic approaches based on histological classifications have now been surpassed by significant advancements in targeting gene mutations specific to lung cancer.^[2,3]^ The molecular categorization of NSCLC plays a crucial role in guiding effective lung cancer therapy. This categorization is based on mutations such as HER2, KRAS, Braf Proto-oncogene (BRAF), and others, which are determined through molecular gene markers and immunohistochemistry.^[4–7]^

Among the various mutations, BRAF mutations have been identified as carcinogenic genes and are found in 3% to 8% of lung tumors. The most prevalent BRAF mutations are BRAFV600E (50%), BRAFG467A/V (35%), and BRAFD549G (6%), listed in descending order. Extensive research has been conducted on BRAF mutations in various cancers, including melanoma, hairy cell leukemia, esophageal cancer, papillary thyroid carcinoma, and serous ovarian cancer.^[8–13]^ Genetic disparities associated with BRAF mutations have been linked to specific clinical and pathological variables, such as smoking habits, gender, neoplastic histology, and clinical stage.^[14,15]^

Despite the presence of BRAF mutations in lung tumors for several years, the connection between BRAF mutations and lung cancer remains a subject of debate due to the scarcity of clinical case studies.^[16,17]^ Consequently, we undertook a comprehensive meta-analysis, examining a significant number of pertinent papers, in order to accurately evaluate the correlation between BRAF mutations and NSCLC.

## 2. Methods

### 2.1. Search strategy and study selection

To identify relevant data, a computer-aided literature search was conducted in the PubMed, EMBASE, ISI Scientific Citation Index, and CNKI databases. The search utilized the following keywords: “BRAF”, “lung carcinoma”, “squamous cell carcinoma”, “lung adenocarcinoma”, and “NSCLC”. The selection criteria encompassed studies that provided free full-text articles, full-text articles, associated data, books and documents, meta-analyses, abstracts, and systematic reviews. Studies such as clinical trials, those lacking relevant data, reviews, and randomized controlled trials were excluded based on predefined standards.

### 2.2. Data collection

Regardless of the qualifying studies, the researchers gathered the following information: the author’s last name, publication year, number of patient cases, country of origin, techniques used for gene detection, count of BRAF mutations, gender, smoking status, histology, and clinical stage of the patients. In case of any discrepancies, all researchers resolved them through discussions.

### 2.3. Statistical analysis

The RevMan application (version 5.3) was utilized to examine the correlation between BRAF mutations and the risk of NSCLC, using odds ratios (OR) and confidence intervals. The chi-square value was employed to evaluate the extent of variability among the included studies. If the chi-square test yielded a *P* value of  < .05, significant heterogeneity was considered present. In such instances, a random effects model was employed. The Begg test was conducted to analyze the potential presence of publication bias. Furthermore, a sensitivity analysis was performed to ensure the consistency of the findings.

## 3. Results

### 3.1. Search outcomes and characteristics

**Figure 1.**
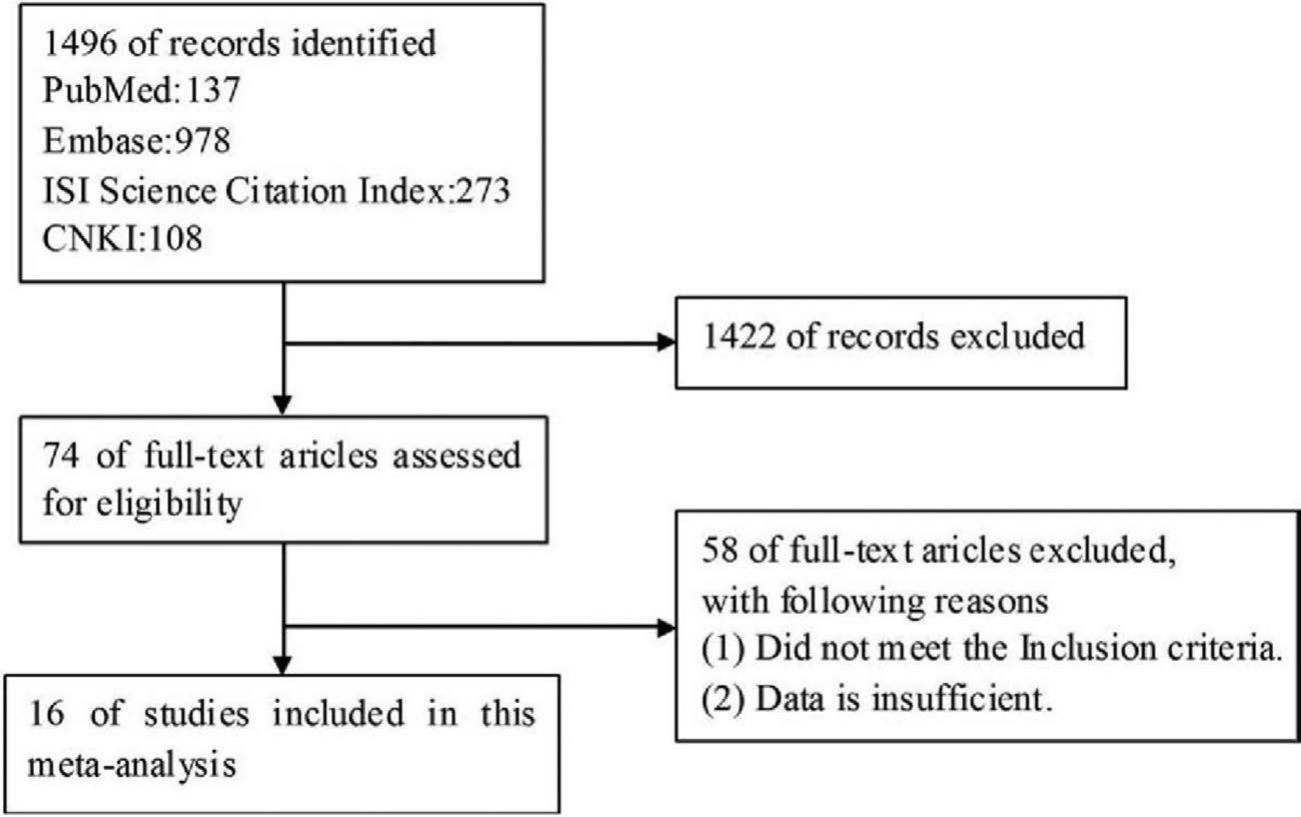
Flowchart of Study Selection Process. A flowchart is presented below, illustrating the stepwise process for the selection of studies to be included in the meta-analysis that investigates the association between BRAF mutation and non-small cell lung cancer. BRAF = Braf Proto-oncogene.

The procedures employed to collect eligible papers for meta-analysis are depicted in Figure 1.

A total of 16 notable and significant major studies were conducted. In this summary, we will focus on a meta-analysis that encompassed a patient cohort of 11,711 individuals. The findings of this meta-analysis are presented in Table 1.^[16,17,19–32,40]^

**Table 1:** Characteristics of the studies included in this meta-analysis.

First author	Year	Source of Pts	Methods	No. of Pts	Mut BRAF (%)	Female (%)	Smokers (%)	ADC (%)	Stage III/IV (%)
Pratilas	2008	4 countries	PCR + SEQ/MALDI-TOF MS	916	17 (1.9)	577 (63.0)	614 (67.0)	623 (68.0)	NA
Schmid	2009	Austria	PCR + SEQ	96	2 (2.1)	38 (39.6)	74 (77.1)	NA	NA
Lee	2010	Korea	PCR + SEQ	173	2 (1.2)	60 (34.7)	117 (67.6)	117 (67.6)	NA
Kobayashi	2011	Japan	PCR + SEQ/SSCP	581	5 (0.9)	204 (35.1)	NA	382 (65.7)	124 (21.3)
Marchetti	2011	Italy	PCR + SEQ/HRMA	1046	37 (3.5)	187 (25.3)	542 (73.3)	739 (70.7)	218 (29.5)
Paik	2011	USA	MALDI-TOF MS	697	18 (2.6)	452 (65.8)	386 (56.2)	NA	NA
An	2012	China	HRMA	452	7 (1.5)	NA	192 (42.5)	307 (67.9)	NA
Sasaki	2012	Japan	PCR + SEQ	305	6 (2.0)	148 (56.7)	NA	NA	NA
Cardarella	2013	USA	PCR + SEQ	883	36 (4.1)	148 (50.5)	229 (78.4)	256 (87.4)	237 (80.9)
Llie	2013	France	PCR + SEQ	450	40 (8.5)	158 (35.1)	403 (89.6)	NA	352 (78.2)
Brustugun	2014	Norway	PCR	979	17 (1.7)	476 (48.6)	NA	646 (66.0)	NA
Kinno	2014	Asian	PCR + SEQ/HRMA	2001	26 (1.3)	935 (46.7)	844 (42.2)	1835 (91.7)	304 (15.3)
Costa	2015	Spain	PCR	80	2 (2.5)	28 (35)	64 (80)	71 (88.7)	NA
Luk	2014	Australia	MALDI-TOF MS	273	7 (2.6)	129 (47.3)	NA	NA	NA
Shao	2015	China	SARMS-PCR	89	1 (1.1)	35 (39.3)	39 (43.8)	88 (98.9)	16 (18.0)
Tissot	2016	France	PCR + SEQ	2690	80 (3.0)	NA	NA	NA	NA

Presents a comprehensive overview of the studies included in the meta-analysis investigating the effects of BRAF mutations in non-small cell lung cancer (NSCLC). The table provides a summary of key characteristics for each study, such as study ID, publication year, study design, sample size, BRAF mutation status, treatment interventions, outcome measures assessed, and other relevant details. This compilation offers valuable insights into the diverse range of studies encompassed within the meta-analysis, enhancing our understanding of the impact of BRAF mutations on NSCLC.

ADC = adenocarcinoma, BRAF = Braf Proto-oncogene, HRMA = high-resolution melting analysis, MALDI-TOF MS = matrix-assisted laser desorption/ionization time of flight mass spectrometry, Mut BRAF = mutant BRAF, NA = not available, PCR = polymerase chain reaction, Pts = patients, sARMS = scorpion probe amplification refractory mutation system, SEQ = sequencing, SSCP = single strand conformation polymorphism analysis.

The relevant articles were identified from November 2008 to January 2016. Among the studies, 6 were conducted in Asia, 6 in Europe, 2 in the United States, 1 in Australia, and 1 in 4 different countries. The stages for including eligible papers in the meta-analysis comprised PCR, PCR + SEQ, and PCR + SEQ/MALDI-TOF MS. Additionally, HRMA and sAMS-PCR techniques were used to detect BRAF mutation.

### 3.2. Characteristic and gene profile

The most prevalent mutations associated with BRAF-altered recurrent fusion (BRAF)-related melanoma in patients include BRAFV600, TP53, and BRAFNon-V600. Numerous studies have demonstrated the clinical and pathological characteristics associated with TP53 and BRAFNon-V600 mutations, despite limited data. In this regard, BRAFV600, TP53, and BRAFNon-V600 mutations were identified in 43%, 19%, and 7% of individuals, respectively.

The occurrence of TP53, BRAFV600, and BRAFNon-V600 mutations was found to be associated with older age and longer overall survival. However, neither TP53 nor BRAFNon-V600 mutations exhibited a significant association with overall survival in relation to first-line tyrosine kinase inhibitor treatment. Similarly, TP53 status did not show any connection with outcomes in first-line BRAF inhibitor therapy.

Although a few patients were excluded from the evaluation of tyrosine kinase inhibitor response due to treatment discontinuation caused by toxicity, most patients experienced disease progression as the primary response to therapy. These findings contribute to our understanding of the clinical characteristics associated with TP53, BRAF, and BRAFNon-V600 mutations in advanced cancer patients. Additionally, they underscore the need for further research into the prognostic significance of TP53 in different groups of cancer patients.

### 3.3. Relationship between BRAF mutations and clinicopathological characteristics in lung cancer

**Figure 2.**
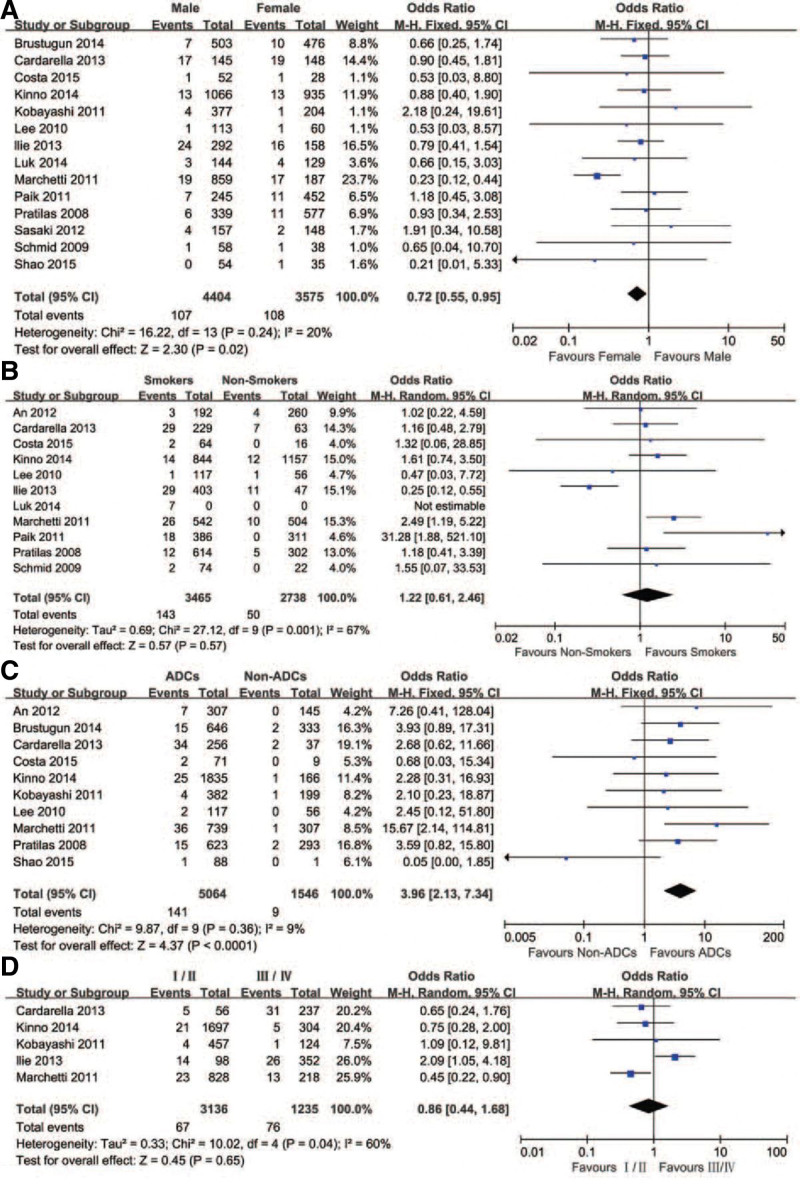
Forest Plot of Overall Effect Size. A forest plot is presented, depicting the pooled effect size estimate (odds ratio) and its corresponding confidence interval for the association between BRAF mutation and non-small cell lung cancer in the meta-analysis. The plot includes individual study-specific effect sizes, providing a comprehensive overview of the research findings. BRAF = Braf Proto-oncogene.

Figure 2 and Table 2 present the associations between BRAF mutations and clinicopathological characteristics in NSCLC. Fourteen studies involving 7979 participants were analyzed to explore the relationship between BRAF mutations and gender. The findings revealed that out of 4404 patients, 107 (2.43%) had BRAF mutations. Among the 3575 female patients, 108 (3.02%) exhibited BRAF mutations. This difference indicated a significant variation in BRAF mutations between males and females (*P* = .02, OR = 0.72, 95% confidence intervals [CI] = 0.55–0.95).

**Table 2:** Relationship between BRAF mutatiinand gendersmoking, histology in NSCLC.

Outcome	Test of association			Heterogeneity test			
	Mutant BRAF (%)	Statistical method	OR (95% CI)	*P* value	X^2^	*I*^2^ (%)	*P* value
Gender							
Male	107/4404 (2.43)	M-H, fixed, 95% CI	0.72 [0.55, 0.95]	.02	16.22	20	.24
Female	108/3575 (3.02)						
Smoking							
Former/current	143/3465 (4.13)	M-H, random, 95% CI	1.22 [0.61, 2.46]	.57	27.12	67	.001
Never	50/2738 (1.83)						
Histology							
ADC	1/5064 (2.78)	M-H, fixed, 95% CI	3.96 [2.13, 7.34]	<.0001	9.87	9	.36
Non-ADC	9/1546 (0.58)						
Stage							
I, II	67/3136 (2.14)	M-H, random, 95% CI	0.86 [0.44, 1.68]	.65	10.02	60	.04
III, IV	76/1235 (6.15)						

This figure legend depicts the findings of a study that investigated the relationship between BRAF mutation and various variables in non-small cell lung cancer (NSCLC). The figure visually represents the associations between BRAF mutation status and gender, smoking status, histology, and stage.

ADC = adenocarcinoma, BRAF = Braf Proto-oncogene, CI = confidence interval, NSCLC = non-small cell lung cancer, OR = odds ratio.

Moreover, eleven studies investigated the connection between BRAF gene mutations and smoking. Among 3465 smokers, 143 (4.13%) showed BRAF mutations, while 50 (1.83%) exhibited BRAF mutations among 2738 nonsmokers. These results indicated no significant difference in BRAF mutations between smokers and nonsmokers (OR = 1.22, 95% CI = 0.61–2.46, *P* = .57).

Additionally, 10 studies explored the association between BRAF mutations and cancer histology. BRAF mutations were detected in 141 out of 5064 adenocarcinomas (ADCs) (2.78%) and 9 out of 1546 non-ADCs (0.58%). This significant difference highlighted the variance in BRAF mutations between ADCs and non-ADCs (OR = 3.96, 95% CI = 2.13–7.34). The relevant data for this analysis were obtained from 5 studies.

Furthermore, the relationship between BRAF mutations and disease stage was assessed in 5 studies. Among 3136 stage I/II NSCLC cases, 67 (2.14%) exhibited BRAF mutations, while 76 (6.15%) showed BRAF mutations among 1235 stage III/IV NSCLC cases. These results indicated no significant difference in BRAF mutations between stage I/II and stage III/IV (OR = 0.86, 95% CI = 0.44–1.68, *P* = .65).

### 3.4. Correlation between BRAFV600E mutation and clinical pathological features in NSCLC

**Figure 3.**
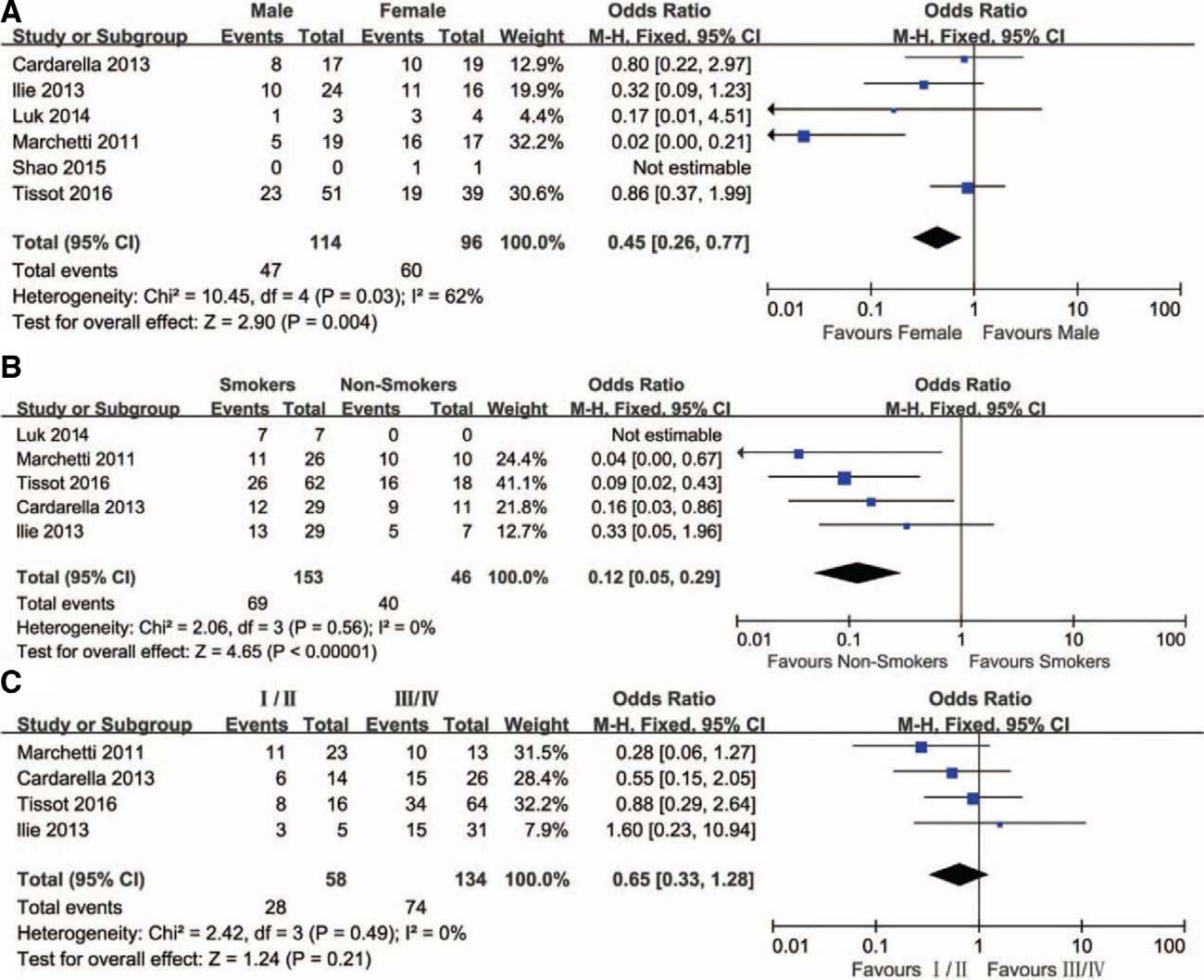
Forest plot depicting the association between BRAFV600E mutations and various factors. (A) Gender: The forest plot displays the odds ratios (OR) and their corresponding confidence intervals (CI) to assess the association between BRAFV600E mutations and gender. The diamond located at the bottom represents the overall effect estimate, while the horizontal line crossing through the diamond represents the confidence interval. (B) Smoking: The forest plot illustrates the odds ratios (ORs) and confidence intervals (CIs) to evaluate the relationship between BRAFV600E mutations and smoking status. Each square on the plot represents an individual study, with the size of the square indicating the study’s weight in the meta-analysis. (C) Stage: The forest plot presents the odds ratios (ORs) and their corresponding confidence intervals (CIs) to examine the association between BRAFV600E mutations and cancer stage. The position of each square on the plot indicates the effect estimate, while the horizontal line represents the confidence interval. The forest plot provides a visual summary of the associations between BRAFV600E mutations and gender, smoking, and stage. It enables an assessment of the overall effect size and the variability among individual studies. BRAF = Braf Proto-oncogene.

Figure 3 illustrates the association between the BRAFV600E mutation and the clinical pathological characteristics in lung cancer. A fixed effects model was employed for a comprehensive analysis. Six studies revealed the presence of BRAFV600E mutations in NSCLC, with 51.0% (107/210) of all BRAF mutations identified as BRAFV600E mutations.

BRAFV600E mutations were detected in 47 (32.6%) out of 144 male patients and 60 (62.5%) out of 96 female patients, indicating a significant disparity in BRAFV600E mutations between genders (OR = 0.45, 95% CI = 0.26–0.77, *P* = .004). Among the patients, 69 (45.1%) out of 153 former or current smokers exhibited BRAFV600E mutations, whereas 40 (87.0%) out of 46 never smokers displayed such mutations. This finding suggests a substantial contrast in BRAFV600E mutations between smokers and nonsmokers (OR = 0.12, 95% CI = 0.05–0.29, *P* < .00001).

Regarding the disease stage, 28 (48.3%) out of 58 patients in stages I or II presented BRAFV600E mutations, while 74 (55.2%) out of 134 patients in stages III or IV exhibited the same mutations. This implies that there is no significant variation in BRAFV600E mutation prevalence between stages I/II and III/IV.

### 3.5. Sensitivity investigation and predisposition of distribution

A thorough sensitivity analysis was conducted to assess the stability of the results, and a Begg funnel chart was generated to evaluate the consistency of the findings. The sensitivity analysis indicated that the exclusion of any of the included variables had no impact on the final outcomes. The funnel plot, presented in Figure 3, exhibited minimal asymmetry, suggesting that the meta-analysis investigating the relationship between BRAF mutations and lung cancer did not demonstrate significant publication bias.

## 4. Discussion

BRAF mutations have been associated with downstream mutational signaling pathways mediated by BRAF, which have garnered significant interest among researchers.^[33]^ Several meta-analyses have been conducted to investigate mutations in papillary thyroid cancer, colorectal cancer, and melanoma, revealing a strong correlation between BRAF mutations and specific pathological parameters such as tumor histology, clinical stage, gender, and smoking status.^[34,36]^ The research findings have shed light on the substantial impact of BRAF mutations on the clinical characteristics of NSCLC. However, due to the limited availability of patients in certain cases, a consensus could not be reached. While a meta-analysis identified a connection between BRAF mutations and NSCLC, the number of cases analyzed was insufficient. Consequently, our revised meta-analysis focused on examining the features of NSCLC patients with BRAF mutations.^[37]^

A total of 16 studies, comprising 11,711 individuals with NSCLC, were included in our systematic evaluation of the relationship between BRAF mutations and NSCLC histology. The BRAF mutation was detected in approximately 2.6% (303/11,711) of the population, which is consistent with previous research.^[24,38]^ The reported rate of BRAF gene mutation ranges from 2% to 5%, similar to the mutation rate observed in our study.^[21,22]^ Our study aimed to enhance the analysis of tumor mutations and examine the characteristics of NSCLC. A meta-analysis was conducted to investigate the relationship between BRAF mutations and 4 clinical and pathological characteristics.

Gender was found to be significantly associated with the BRAF mutation rate (OR = 0.72, 95% CI = 0.55–0.95, *P* = .02). Specifically, a subgroup of BRAF mutations was identified in women with colorectal cancer.^[37]^ Additionally, we observed a correlation between BRAF mutations and smoking. NSCLC is classified into 3 histological types: ADC, large cell carcinoma, and squamous cell carcinoma, with ADC accounting for over half of all cases. Our meta-analysis revealed that BRAF mutations are more prevalent in ADCs compared to other histological types (OR = 3.96, 95% CI = 2.13–7.34, *P* < .0001), which is consistent with previous research.

Clinical stage plays a crucial role in determining the prognosis of NSCLC. However, our meta-analysis did not discover any significant associations between BRAF mutations and the stage of NSCLC. This outcome may be attributed to the limited number of available patient cases. The most prevalent mutation in the BRAF gene is BRAFV600E40. To date, 6 studies have investigated the relationship between clinicopathological characteristics and the BRAFV600E mutation.^[19,21,27,30–32]^ These studies revealed significant differences in the clinical characteristics of NSCLC patients with and without BRAFV600E mutations.^[19,21,27]^ While several studies reported a higher prevalence of BRAFV600E mutations in women and nonsmokers, this association was not observed in other clinicopathological aspects.^[21,27]^[Bibr R18][Bibr R35][Bibr R39][Bibr R41]

In our meta-analysis, we found that the BRAFV600E mutation is more frequent in women than in males. Additionally, the BRAFV600E mutation was significantly more prevalent in nonsmokers compared to smokers (OR = 0.12, 95% CI = 0.05–0.29, *P* < .00001) [27]. We also observed a correlation between non-BRAFV600E mutations and early-stage cancers, although statistical significance was limited, likely due to the scarcity of cases. Several limitations of our study should be acknowledged when interpreting the findings, including insufficient studies focusing on BRAFV600E mutations. Therefore, further research is needed to expand and validate our results. Additionally, due to the lack of data, the association of BRAF mutations with clinical stage is not well elucidated.

Lastly, we discovered a significant association between BRAF mutations in NSCLC patients and a predisposition to ADC in women. Furthermore, the BRAFV600E mutation was linked to NSCLC in both women and nonsmokers. These findings provide a theoretical basis for the diagnosis of NSCLC, especially considering smoking status, which aligns with previous investigations.

**Figure 4.**
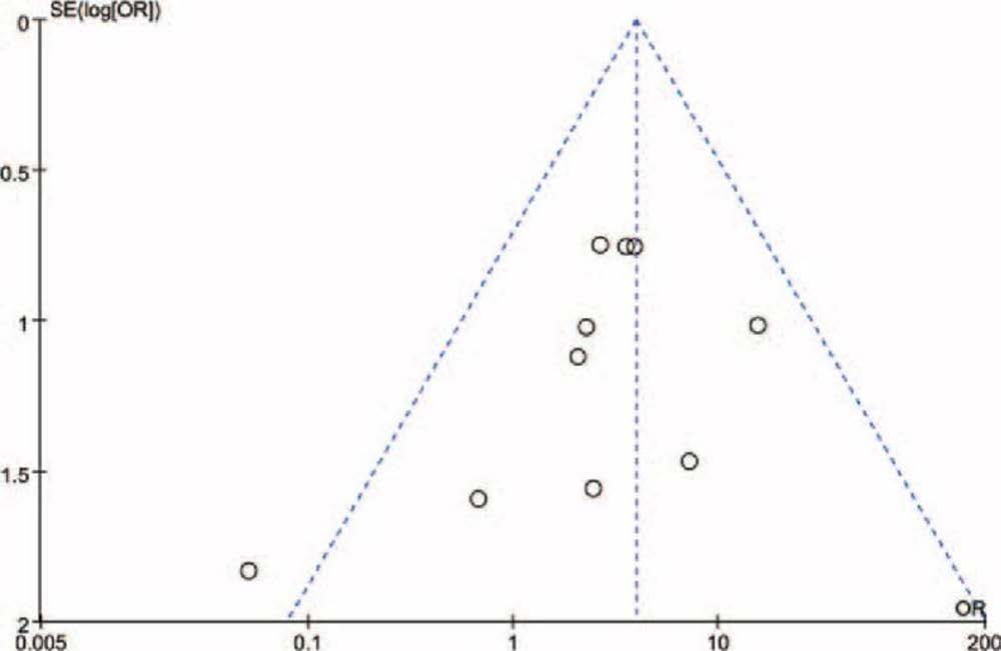
Begg Funnel Plot for Publication Bias of BRAF Mutations and ADCs Risk. This plot illustrates a Begg funnel plot used to evaluate the presence of publication bias in studies investigating the correlation between BRAF mutations and the risk of Adverse Drug Reactions (ADRs). The Begg funnel plot serves as a graphical representation employed for examining publication bias, primarily in meta-analyses or systematic reviews. The plot visually portrays the connection between effect sizes or treatment effects and their corresponding standard errors in individual studies. In this analysis, the plot is utilized to assess the distribution asymmetry of the studies, which can indicate the potential presence of publication bias. If the plot demonstrates a symmetrical distribution, it suggests a low risk of publication bias. Conversely, an asymmetrical distribution may suggest the existence of bias. ADCs = adenocarcinomas, BRAF = Braf Proto-oncogene.

## Acknowledgments

We are sincerely grateful to all the members of The First Oncology Department at Jinzhou First Affiliated Hospital for their invaluable contributions through insightful discussions and unwavering support. We extend our heartfelt appreciation to Professor Xiaomei Liu for her innovative ideas and design concept. Furthermore, we express our deep gratitude to Li Xi for providing valuable statistical advice, which greatly enhanced the quality of our work.

## Author contributions

**Conceptualization:** Liu Xiaomei.

**Data curation:** Clint Taonaishe Chimbangu, Li Xi.

**Formal analysis:** Clint Taonaishe Chimbangu.

**Funding acquisition:** Clint Taonaishe Chimbangu.

**Investigation:** Clint Taonaishe Chimbangu.

**Methodology:** Clint Taonaishe Chimbangu.

**Project administration:** Clint Taonaishe Chimbangu.

**Resources:** Clint Taonaishe Chimbangu, Zhou Ya, Yu Xingxu, Zhao Jiayue, Meng Xiao, Wang Ying.

**Software:** Clint Taonaishe Chimbangu.

**Supervision:** Clint Taonaishe Chimbangu.

**Validation:** Clint Taonaishe Chimbangu, Yu Xingxu, Zhao Jiayue, Meng Xiao, Wang Ying.

**Visualization:** Clint Taonaishe Chimbangu.

**Writing – review & editing:** Clint Taonaishe Chimbangu.
